# One-Dimensional Nanocomposites Based on Polypyrrole-Carbon Nanotubes and Their Thermoelectric Performance

**DOI:** 10.3390/polym13020278

**Published:** 2021-01-16

**Authors:** Neazar Baghdadi, M. Sh. Zoromba, M. H. Abdel-Aziz, A. F. Al-Hossainy, M. Bassyouni, Numan Salah

**Affiliations:** 1 Center of Nanotechnology, King Abdulaziz University, Jeddah 21589, Saudi Arabia; nebaghdadi@kau.edu.sa (N.B.); nsalah@kau.edu.sa (N.S.); 2 Department of Chemical and Materials Engineering, King Abdulaziz University, Rabigh 21911, Saudi Arabia; mhmossa@kau.edu.sa; 3 Department of Chemistry, Faculty of Science, Port Said University, Port-Said 42521, Egypt; 4 Department of Chemical Engineering, Faculty of Engineering, Alexandria University, Alexandria 21544, Egypt; 5 Chemistry Department, Faculty of Science, Northern Border University, Arar 1321, Saudi Arabia; ahmed73chem@scinv.au.edu.eg; 6 Chemistry Department, Faculty of Science, New Valley University, Al-Wadi Al-Gadid, Al-Kharga 72511, Egypt; 7 Department of Chemical Engineering, Faculty of Engineering, Port Said University, Port Fouad 42511, Egypt; 8 Materials Science Program, University of Science and Technology, Zewail City of Science and Technology, October Gardens, 6th of October, Giza 12578, Egypt

**Keywords:** polypyrrole, one-dimensional nanocomposites, carbon nanotubes, thermoelectric properties, conducting polymers

## Abstract

Conducting polymers have attracted significant attention due to their easy fabrication, morphology modification, and their electrical properties. Amongst them, polypyrrole (PPy) has attractive thermoelectric (TE) properties. Engineering of this polymer in one-dimensional (1D) nanostructured form is found to enhance its TE performance. This was achieved in the present work by using multi-walled carbon nanotubes (MWCNTs) as a core template to direct the self-assembly of PPy and also to further enhance its TE performance. The growth of PPy on the sidewalls of MWCNTs was performed in an acidic medium based oxidative in situ polymerization. Various concentrations of MWCNTs within the range 1.1–14.6 wt.% were used to form the MWCNTs/PPy nanocomposites in 1D core-shell structures. The morphology and microstructure results of the produced nanocomposite samples showed that this MWCNTs were successfully coated by thick and thin layers of PPy. At low concentrations of MWCNTs, thick layers of PPy are formed. While at high concentrations thin layers are coated. The formed 1D nanocomposites have enhanced TE performance, particularly those containing higher contents of MWCNTs. The power factor and figure of merit values for the formed 1D nanocomposites recorded around 0.77 µV/mK^2^ and 1 × 10^−3^ at room temperature (RT), respectively. This enhancement was attributed to the perfect coating and good interaction between PPy and MWCNT through π–π stacking between the polymer chains and these nanotubes. These results might be useful for developing future TE materials and devices.

## 1. Introduction

Recently, organic materials as thermoelectric (TE) mainly including conducting polymers and their composites have observed fast progress, this is due to their various advantages relative to the traditional inorganic thermoelectric materials, such as solution processability, low cost, light-weight, low thermal conductivity (k), and good flexibility. For instance, by controlling combination with carbon nanotubes (CNTs) or graphene nanosheets organic polymers have been established to gain significant enhancements in their thermoelectric performance [1,2,3,4]. Until now, the main polymers that have been used as thermoelectric materials are conducting polymers, including poly(3,4-ethylene dioxythiophene) (PEDOT) [5,6,7], polyaniline (PANI) [8], polypyrrole (PPy) [9]. However, the interaction between carbon nanomaterials and such polymers should be carefully addressed. In the absence of a good interaction, there will be limited enhancements in the desired properties [10,11], therefore choice of a suitable conducting polymer along with the appropriate starting compounds are of great importance.

Among the above-mentioned polymers, polypyrrole (PPy) is a potential thermoelectric (TE) material, mostly because it has a low thermal conductivity and high electrical conductivity, especially when doped with different suitable dopants. Besides, PPy has many advantages, such as being easy to fabricate, low-density, and having good mechanical properties. Consequently, more researchers have focused on the thermoelectric properties of neat PPy or PPy composites [12]. Composite formation was adopted to improve the TE performance of a material, which is evaluated by its dimensionless figure of merit, $zT=\;\alpha^{2}\sigma /k$, where *T*, *α*, *σ*, and *k* are the absolute temperature, Seebeck coefficient, electrical conductivity, and *k* thermal conductivity, respectively. To obtain high *zT*, the thermoelectric material should have a high Seebeck coefficient, electrical conductivity, and a low thermal conductivity. It is very hard to control these three parameters at the same time to get a high *zT* value because they are depending on each other [13].

There are some attempts were tried to form CNTs/PPy composites in a core shell structure, however perfect coating could not be achieved. For example, the work reported by Wang et al. [14], who produced MWCNTs/PPy nanocomposites using p-toluenesulfonic acid (TSA) as a dopant and iron chloride (FeCl_3_) as an oxidant was focused on producing core shell structures. They obtained PPy nanoparticles at the surface of the MWCNTs, without forming a uniform coating [14]. It is therefore quite important to find out suitable approach and proper percussors that can produce core shell structures of CNTs/PPy, preferably in one dimensional (1D) with perfect smooth coating. Such 1D core shell structure with a perfect coating might be the key factor to facilitate the charge transport and to improve the electrical conductivity. This also might assist on forming energy filtering sites at the interfaces between PPy and CNTs, which can improve or even maintain the Seebeck coefficient value.

In this work, polypyrrole has been synthesized in the presence of sodium dodecyl sulfate (SDS) as a surfactant to control its morphology as a blank thermoelectric polymer. Subsequently, multi-walled carbon nanotubes/PPy (MWCNTs/PPy) with different MWCNTs loadings have been synthesized using in situ polymerization method. The as produced PPy/MWCNTs composites have been characterized by several well-known techniques such as SEM, TEM, Raman, FTIR, and XPS spectroscopy. Then, their TE performance was evaluated and the obtained results are discussed in more details.

## 2. Experimental

### 2.1. Materials

Pyrrole, sodium dodecyl sulfate (SDS), ethanol, and anhydrous ferric chloride. The reagents used were purchased from Sigma-Aldrich, Steinheim, Germany and were of analytical grade (99.99%). Industrial grade multi-walled carbon nanotubes (MWCNTs) were used with the following specifications: Diameter 10–40 nm, length 1.5 µm, carbon purity 90%, metal oxide, ≈10% and BET surface area 250–300 m^2^/g.

### 2.2. Synthesis of MWCNTs/PPy Nanocomposites

The preparation of MWCNTs/PPy nanocomposites was performed as follows: 3.5 g sodium dodecyl sulfate (SDS) was dissolved in 100 mL absolute ethanol and then the solution was diluted by water up to 400 mL using a magnetic stirrer (850 rpm) at room temperature. The following weights of multi-walled carbon nanotubes, 0.05, 0.10, 0.20, 0.40, and 0.80 g were separately added to each SDS solution at previous conditions for 20 min. Four (mL) pyrrole monomer was added to each dispersion. Then each dispersion was shifted to an ultrasonic homogenizer for 20 min. 160 mL (0.5 M FeCl_3_) was added drop by drop to the resulting dispersions after using an ultrasonic homogenizer for 45 min. After finishing the addition of initiator (FeCl_3_), the resulting dispersions has been left under magnetic stirrer for an additional 1 h and then were left overnight. The filtration process was carried out using distilled water followed by ethanol. The resulting composites were dried at 60 °C for 2 days and marked by PN1, PN2, PN3, PN4, and PN5 as displayed in Table 1.

**Figure 1:**
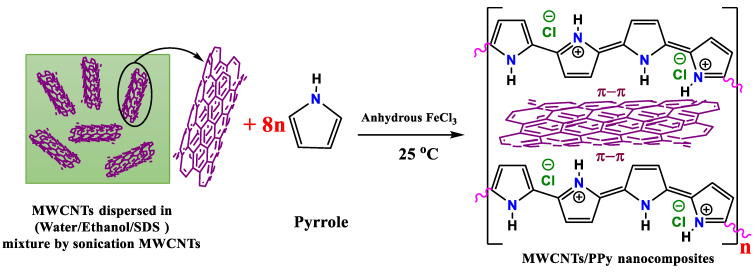
Preparation scheme of polypyrrole/MWCNTs nanocomposites.

The preparation scheme of MWCNTs/polypyrrole (PPy) nanocomposites is displayed in Figure 1.

### 2.3. Characterization and TE Measurements

Polypyrrole, MWCNTs, and MWCNTs/PPy morphologies were examined with the aid of scanning electron microscopy (SEM) (JSM-7500F, JEOL, Tokyo, Japan) and transmission electron microscopy (TEM) (JEM 2100F, JEOL). On a micro-Raman spectroscope, the Raman spectra were recorded (Thermo Fisher Scientific, Boston, MA, USA). The FTIR spectra of the samples were derived from a slow-modified reflection. Total reflection Fourier-transform infrared (Thermo Fisher Scientific) spectroscopy. Samples were measured on the PHI 5000 Versa Probe, Japan, through radiographic photoelectron spectroscopy (XPS).

A hydraulic manual press was used to prepare pellets for the pure and MWCNTs/PPy nanocomposites. Around 7 tons was applied using the hydraulic press to make pellets with 13 mm in diameter and ranged in thickness from 1 to 2 mm. The pellets were rinsed in a vacuum oven at 440 K for one hour. The resistivities and Seebeck coefficients were calculated to analyze the electrical and thermal properties of the pellets. In helium atmosphere, the resistivities and Seebeck coefficients of the pellets were calculated using the computerized LSR-3 Linseis–Seebeck coefficient and electrical resistivity system (Linseis, Selb, Germany). The dimensions of the pellets introduced in to the system to calculate the resistivity, while the heating rate and temperature difference between the hot and cold sides were set at 5 °C/min and 50 °C, respectively. The recorded temperature gradient was in the range 4–6 °C for these samples. The corresponding Seebeck voltage values were recorded by the system and the values of the Seebeck coefficient were automatically calculated and saved by the LSR-3 system. For calculating the thermal conductivity of the samples, a laser flash-thermal conductivity analyzer (LFA-1000, Linseis, Selb, Germany) was used. The dimensions were perpendicular to the pellet surface.

## 3. Results and Discussion

### 3.1. Structural Properties

**Figure 2:**
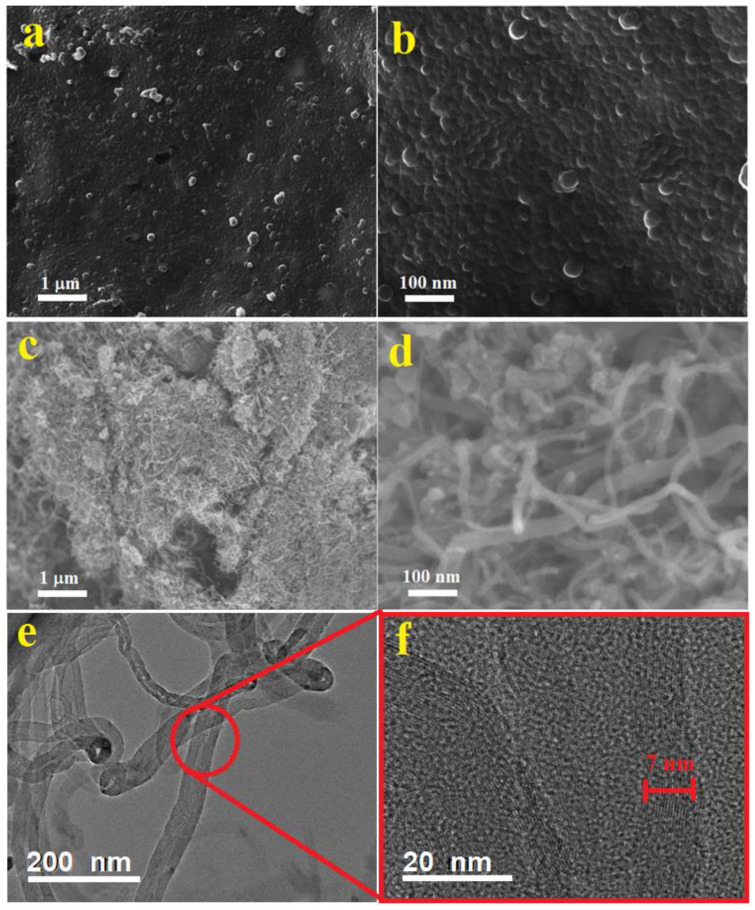
SEM images at two different magnifications of pure PPy (**a**,**b**), MWCNT (**c**,**d**). TEM (**e**) and HRTEM (**f**) images of MWCNTs are also shown.

The surface morphology and nanostructure form of the synthesized pure PPy were investigated by SEM technique. The obtained SEM images are represented in Figure 2a,b. These SEM images show that the PPy structures is produced in a 2-dimensional film. These films are composing fine nanoparticles with sizes in the range 15–25 nm. The shape and size of the resulting polymer is well-known to depends on the type of used surfactant during the polymerization process. The formation of PPy films in the presence of SDS and FeCl_3_ was reported by Gangopadhyay [15] (dissolved in a water medium), but it is stated that the films are too brittle to handle freely. This perhaps might be due to the absence of ethanol as a co-solvent, or may be due to the variations in the molar ratios of the pyrroles and surfactants/oxidants, which might affect the bonding between the formed PPy chains.

Figure 2c–e shows SEM and TEM images of the MWCNTs at different magnifications. Thin and thick nanotubes can be observed in bundle entangled forms (c). The diameters of these MWCNTs are in the range 10–40 nm. These nonuniform MWCNTs were used as received without further purification or catalysts removal, therefore a considerable defects and catalyst particles are noticeable in these nanotubes (Figure 2d,e). The HRTEM image shows multi-walled nanotubes of around 15–18 walls with walls thicknesses around 7 nm as shown in Figure 2f. Zigzagged walls can be observed in the thin and thick MWCNTs. Moreover, bamboo like structures is also noticed in some of the thin MWCNTs (e).

**Figure 3:**
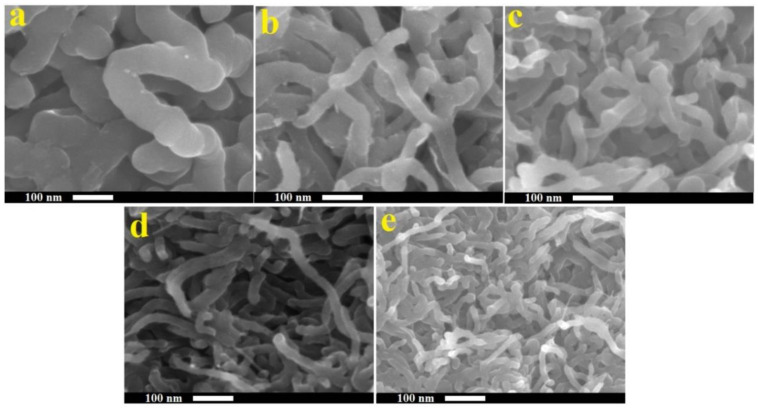
SEM images at the same magnification of MWCNTs/PPy nanocomposites ((**a**): PN1, (**b**): PN2, (**c**): PN3, (**d**): PN4, (**e**): PN5).

**Figure 4:**
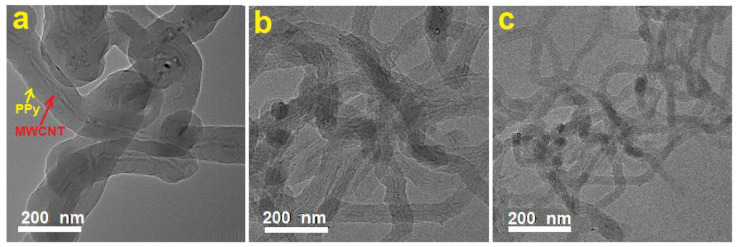
Typical TEM images at the same magnification of MWCNTs/PPy nanocomposites ((**a**): PN1, (**b**): PN3, (**c**): PN5).

The morphology and microstructure of the MWCNTs/PPy composites were investigated by the SEM and TEM and the obtained results are presented in Figure 3 and Figure 4, respectively. It is clear that the PPy were grown on the side walls of these MWCNTs, making one dimensional (1D) nanocomposite. Perfect coating can be observed, but at different thicknesses (Figure 3). Lower amounts of MWCNTs results on thicker shells/layers of PPy, while higher amounts of MWCNTs could produce thinner layers of PPy. These observations are clearly shown in Figure 4. The coating was performed with different ratios between PPy and MWCNTs as mentioned in Table 1. It is clear that with increasing the percentage of MWCNTs, the PPy layer thicknesses were decreased. As mentioned above the amount of Py was fixed, while that of MWCNTs vary from 1.1 to 14.6 wt.%. This perhaps could distribute the available monomers of PPy on the surface of MWCNTs, therefore the higher amounts of MWCNTs got thin layers of PPy. This results in forming MWCNTs/PPy nanocomposites with different diameters as shown in Figure 3a–e.

Figure 4 shows TEM images at the same magnification of typical MWCNTs/PPy nanocomposites (a: 1.1 wt.%, b: 4.6 wt.%, c: 14.6wt.%). In this figure, it is clear that the MWCNTs are located at the cores of these 1D structures, and the PPy are forming the shells. With increasing the percentage of MWCNTs, the thickness of the coated PPy decreases and vice versa as illustrated in Figure 4a–c. This result is remarkable, by means it is possible that the presence of SDS as a surfactant could favor the PPy growth smoothly in the side walls of MWCNTs, which themselves acted as 1D templates. This result is much better than that reported earlier [14] on producing MWCNTs/PPy nanocomposites using p-toluenesulfonic acid (TSA) as a dopant and iron chloride (FeCl_3_) as an oxidant. They obtained PPy nanoparticles at the surface of the MWCNTs, without a uniform coating [14]. It is clear that the used surfactant in our case is the key factor on obtaining 1D core/shell MWCNTs/PPy nanocomposites.

**Figure 5:**
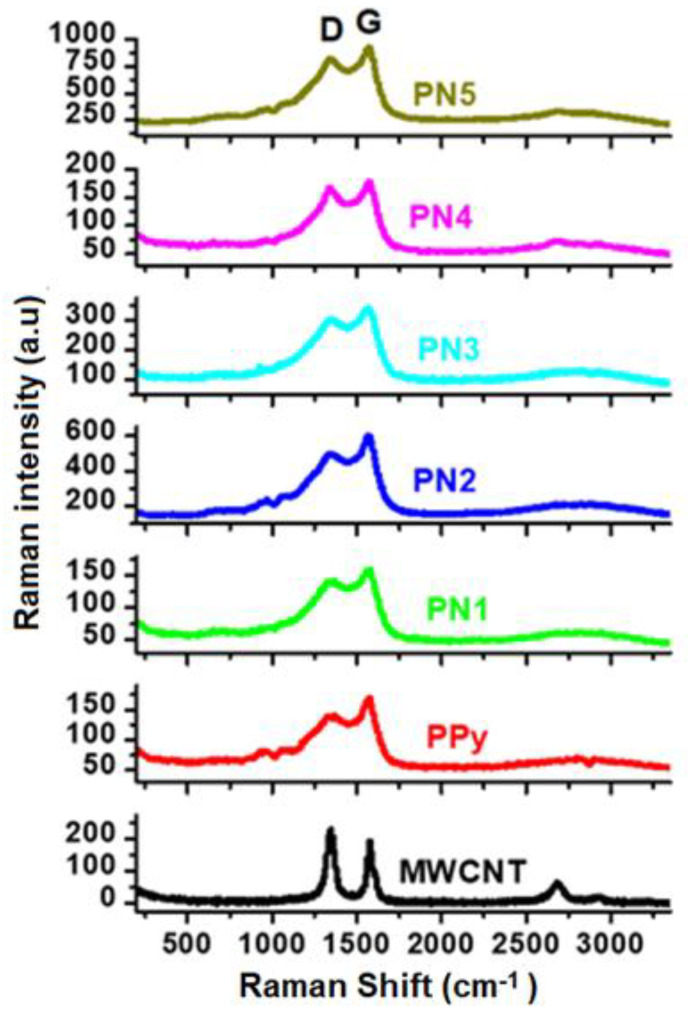
Raman spectra of the MWCNTs/PPy nanocomposites. Spectra of the pure MWCNTs and PPy are also shown.

Figure 5 displays the Raman spectra of Nanocomposites MWCNTs, PPy and PPy/MWCNTs. 5. Two identified bands of 1598 and 1345 cm^−1^ were seen in the neat MWCNTs. These are the vibration of the MWCNTs (G band) caused by the condition (D-band) and in-plane vibration. There were two large bands with a duration of approx. 1362 and 1571 cm^−1^ corresponding to the vibration modes C–C and C=C in the Ring of the PPy backbone [16,17] respectively. Weak bands of 952 and 1081 cm^−1^ were also present in the neat PPy. Both are the in-plan deformation of the ring associated with the di-polaron and C–H respectively [18]. The MWCNTs/PPy spectrum is similar to the neat PPy spectrum, but the range of amplitude increases systematically by increasing the MWCNTs material. Furthermore, there is a shift in the composites from the 1571 cm^−1^ tidy PPy band to the higher wave number line. This increase and band change means that MWCNTs are associated with PPy chains by moving the charge. A major decrease in the intensity of the band has contributed to a successful π–π stacking between the MWCNTs and PPy chains for composites with the smaller percentage of MWCNTs [19].

**Figure 6:**
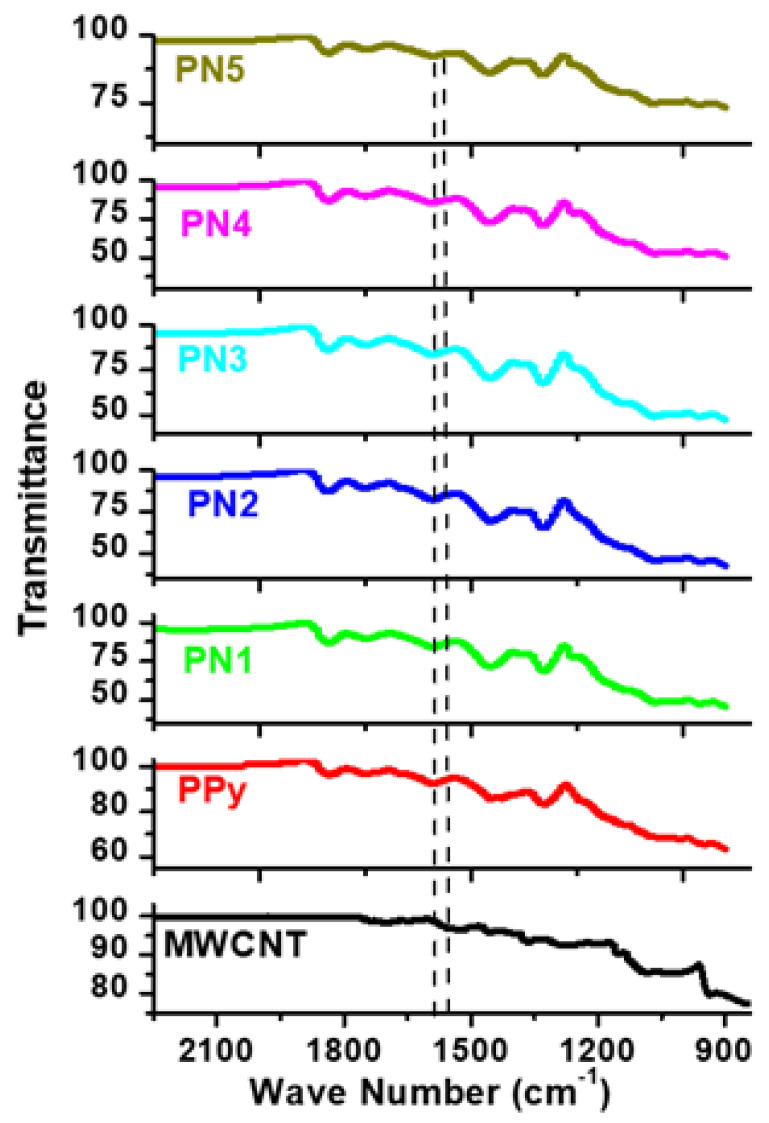
FTIR spectra of the MWCNTs/PPy nanocomposites. Spectra of the pure MWCNTs and PPy are also shown.

FTIR spectra for PPY, MWCNTs, and /MWCNTs/PPy nanocomposites are presented in Figure 6. This chart shows 1550 and 1450 cm^−1^ bands. These fit the stretching pulse of C=C and C–N of PPy. The vibration of the pyrrole ring formed the band at 1170 cm^−1^. The in-plane vibratory deformation of C–H and N–H occurred at 1040 cm^−1^ and the out-plane deformation band at 860 cm^−1^ [20]. The 1130 cm^−1^ band is PPy (doped with chloride ion) characteristic [21]. Due to the overlap with the vibration of the pyrrole ring at 1170 cm^−1^ the expected S=O expanded vibration at 1183 cm^−1^ could not be detected [22]. The PPy/MWCNTs composites with PPy have an FTIR spectrum similar to the neat MWCNTs and PPy. This confirms the existence in the composites of all characteristic bands of PPy and MWCNTs. With a growing percentage of MWCNTs, the intensity of the C–H band shifted considerably. This is can be credited to the π–π assembling MWCNTs and PPy backbones.

**Figure 7:**
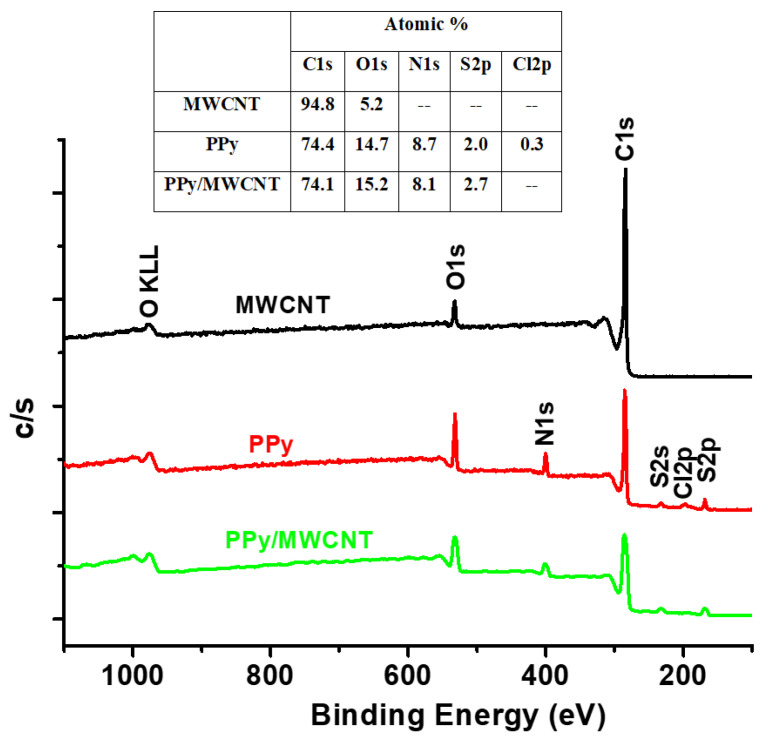
Typical XPS survey profiles of MWCNTs, PPy, and MWCNTs/ PPy nanocomposites (PN2). The XPS elemental analysis and their atomic percentage are also shown in the table.

**Figure 8:**
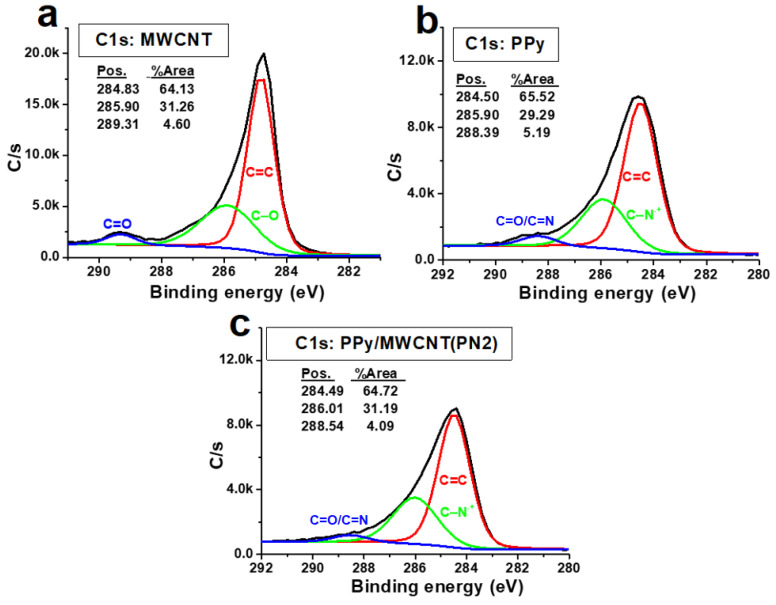
C1s XPS profiles of (**a**) MWCNTs, (**b**) PPy, and (**c**) MWCNTs/ PPy nanocomposites (PN2).

The XPS survey profiles of the MWCNTs, PPy, and MWCNTs/PPy nanocomposites are shown in Figure 7. The C1s and O1s OKLL peaks could be observed in the spectra of the neat of both MWCNTs and PPy. A weak Cl2p and S2P bands were observed at around 200 eV for both neat PPy and PPy/MWCNTs composite. These peaks are due to the chloride ion-doped from FeCl_3_ initiator and sulfur gained from SDS surfactant during the polymerization process. The C1s/O1s ratio of the neat CNTs is higher than that of neat PPy. The intensity of the O1s band decreased slightly for the PPy/MWCNTs composites with the appearance of a weak specified peak for chloride ion (Cl2p band at around 200 eV). This indicates the success of doping with chloride ions for the PPy chains. The C1s XPS profiles of MWCNTs, PPy, and MWCNTs/ PPy nanocomposites (PN2) are shown in Figure 8(a-c). As shown in Figure 8b, the observed peaks at 284.5 eV and 285.90 eV, which occupied areas of 65.52%.and 29.29% might be attributed to hybridization of carbon sp^2^ and sp^3^ type, respectively. The peak at 288.6 eV that occupied an area of 5.9% might be due to C-N of PPy. As shown in Figure 8c, the observed three peaks at 284.49, 286.01 and 288.5 eV are occupied areas of 64.72%, 31.19%, and 4.09%, respectively. The C=C band is the most intense one in the spectrum of neat PPy; however, its intensity is lower than that of the C=C band in the MWCNTs (Figure 8a). The spectra of the coated MWCNTs by PPy in the current composite (2.17% MWCNTs) is nearly similar to the spectra of the uncoated MWCNTs and neat PPy; though, the intensity of the resulting band of the composite to that of the neat MWCNTs and PPy (lower than that of the pure MWCNTs but higher than that of PPy). There are no significant changes are observed in the peak positions. This confirms that MWCNTs have been successfully coated by PPy with no extra bonds or crosslinking in the contact surfaces. The excellent π–π stacking between PPy backbones and MWCNTs might also assist in stabilizing.

### 3.2. Thermoelectric Properties

**Figure 9:**
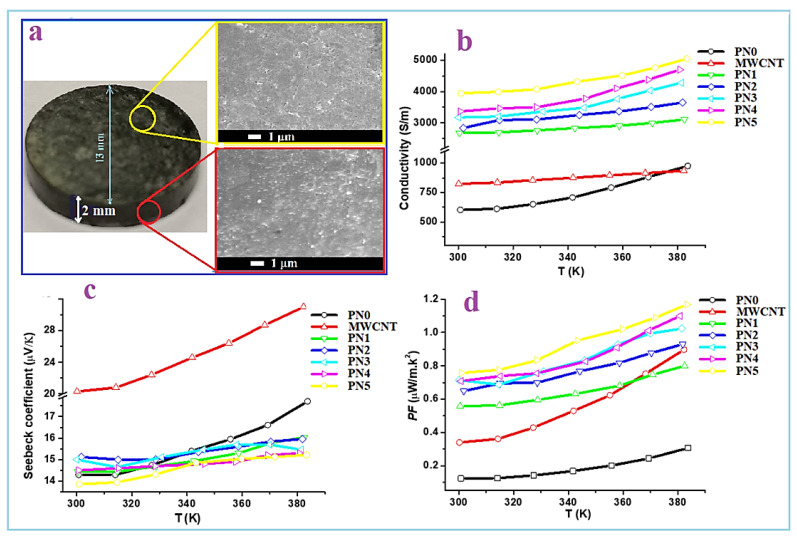
(**a**) Picture of a typical MWCNT/PPy pellet (PN3) along with its SEM top and cross section views. (**b**–**d**) Thermoelectric (TE) performance of the PPy/MWCNT nanocomposites as the functions of temperature.

To investigate the TE properties of the above mentioned MWCNTs/PPy nanocomposites powder samples, they were initially pressed in pellets forms as described in the experimental section. Figure 9a shows a picture of one of the formed MWCNTs/PPy pellets, which is of the PN3 sample. SEM images with a top and cross section view of this pellet are also shown. These images show well pressed pellet with almost no formation of cracks or defects. The produced 1D nanocomposites of MWCNTs/PPy were easily aligned under the applied press (~7 tons) making solid pellet with a good contact between its contents. The top and cross section view SEM images clearly show highly dense pellet with side-to-side contact of the 1D nanocomposite. This is an essential requirement to facilitate the charge transport across the whole pellet.

The TE properties of the pure PPy (PN0), neat MWCNTs and MWCNTs/PPy nanocomposites (PN1-PN5) are shown in Figure 9b–d. The measured electrical conductivity at room temperature for PPy is around 600 S/m, while it is approximately 830 S/m for MWCNTs Figure 9b. These values increased with an increase in temperature up to 383 K reaching to 930 S/m for the PPy and 900 S/m for MWCNTs. The relatively low electrical conductivity of the MWCNTs used in this study as compared to those of the MWCNTs reported in the literature [23] might be attributed to their microstructure, as shown in Figure 2c–f. The zigzagged structure and bamboo-like form of these MWCNTs in addition to the considerable amounts of defects/metal oxides are greatly affecting the electrical conductivity. The poor graphitization of the used MWCNTs (as reflected form the low ratio of G to D bands of Raman, shown in Figure 5) is also another reason for the low electrical conductivity. These are responsible for hindered the mobility of charge carriers, thus reducing the electrical conductivity. It is well known that oxygen containing graphitic carbon materials are p-type semiconducting [24]. In the present MWCNTs, the XPS results shown in Figure 7 and Figure 8 display limited amount of oxygen which might not be enough to enrich the MWCNTs with carrier holes, and thus showed only low electrical conductivity. In case of the PPy, its electrical conductivity is comparable to or even higher than those with similar structures or morphologies as reported by other research groups [25,26].

The measured electrical conductivity as a function of temperature of the MWCNTs/PPy nanocomposites (PN1-PN5) shown in Figure 9a increased with an increase in the MWCNTs content. For PN0 with the rise in temperature, some electrons gain energy and then become free to conduct electricity (PPy is well known as a good conducting polymer). Therefore, polypyrrole’s electric conductivity increases with temperature. The increased conductivity of MWCNTs with temperature can be attributed to the decreasing in the contact resistance with temperature. At room temperature, the electrical conductivity was recorded around 2700 S/m for the MWCNTs/PPy nanocomposite at a MWCNTs content of 1.1 wt.% (PN1). This value was increased to approximately 4000 S/m by increasing the MWCNTs content to 14.6 wt.%. The measurements demonstrated that the electrical conductivity of the MWCNTs/PPy nanocomposites showed a semiconductor behavior, e.g., an increasing trend with an increase in temperature. At 383 K, the electrical conductivity values of all the nanocomposite samples were 15–25% higher than those recorded at room temperature. This significant enhancement in the electrical conductivity of the nanocomposites is remarkable, which might be attributed to the smooth and perfect PPy coating formed on the surface of the MWCNTs. Agglomerations of PPy or particles forming on the side walls of these nanotubes was not observed as shown in the SEM and TEM images.

Excellent 1D lamination may promote the shipment of charges and maximize the concentration of freight easily by incorporating both MWCNTs and PPy carriers. The results shown for PPy nanowire/graphene composites are compatible with previous works [26]. In addition, the increase in the power conductivity of MWCNTs/PPy composites has been recorded in the literature [14] and it is due to MWCNTs serving as a template to direct PPy’s self-assembly through the β-та interplacement of PPy with MWCNTs during production. In the formed layers and especially at interfaces, the concentration of the available transporters can be high, thereby improving electrical conductivity, with the effect on load mobility. The interfaces layers are higher in case of higher MWCNTs contents, therefore showing enhanced electrical conductivity. As temperature increases, some electrons acquire energy and then become free for electrical conduction. Hence, the electrical conductivity of polypyrrole increases with temperature.

The measured Seebeck coefficient of the pure PPy (PN0), MWCNTs and MWCNTs/PPy nanocomposites (PN1-PN5) are shown in Figure 9b. In case of pure PPy, it has Seebeck value around 14.5 µV/K at RT, while the neat MWCNTs has a value of 20 µV/K. These values were increased by around 30% with an increase in the temperature to 383 K. These values for both PPy and MWCNTs are close to those reported previously [19]. In case of the MWCNTs/PPy nanocomposites, the obtained Seebeck coefficient values are almost similar to that of the pure PPy. But these values are much higher than that reported for similar composite [14]. As a result of the improved electrical conductivity and almost invariant Seebeck coefficient, the calculated power factor, *PF* values of the nanocomposite samples are higher than those of the individuals PPy and MWCNTs as displayed in Figure 9c. The maximum value is recorded by the nanocomposites of PN5 at MWCNTs content of 14.6 wt.%. It recorded 0.77 µW/mK^2^ at room temperature, and this value increased to 1.3 µW/mK^2^ at 383 K. Although the MWCNTs has low electrical conductivity as compared to those reported in the literature [23], the *PF* value of the composite samples are higher than that of MWCNTs/PPy composites [14].

**Figure 10:**
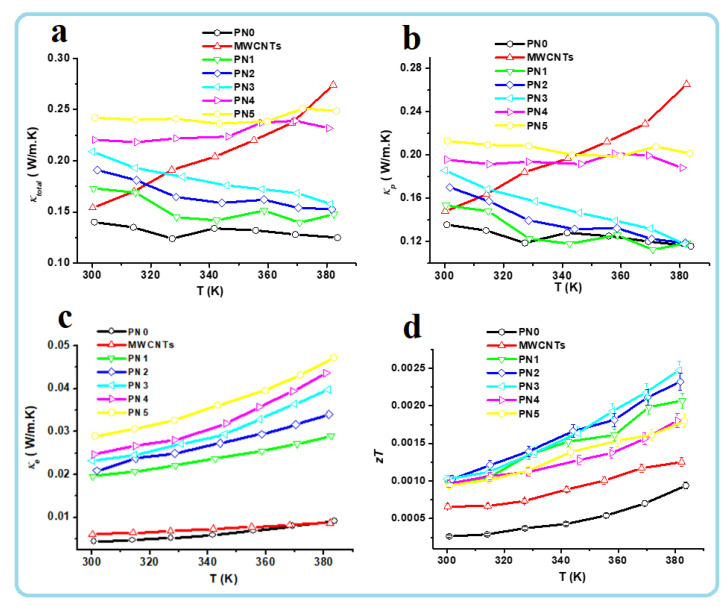
Total thermal conductivity, *κ*_total_ (**a**), phonon thermal conductivity, *κ*_p_ (**b**), electron thermal conductivity, *κ*_e_ (**c**) and figure of merit, *zT* (**d**) of the MWCNTs/PPy nanocomposites as the functions of temperature.

It is well known that in solid materials, heat is transferred mainly by phonons and electrons, so the total thermal conductivity is defined as *κ*_total_= *κ*_p_ + *κ*_e_, where *κ*_p_ and *κ*_e_ are the phonon and the electron thermal conductivities, respectively. The value of *κ*_e_ can be determined using the measured electrical conductivity σ and then the Wiedemann–Franz law on the assumption that the relaxation time of phonons and electron holes is identical [27].

$$L=\frac{k_{e}}{\sigma T}=\frac{\pi^{2}k_{B}^{2}}{3e^{2}}=2.44\;\times 10^{-8}\;WΩk^{-2}$$

where *L* is the Lorenz number, *k_B_* is Boltzmann’s constant, and *e* is the charge of an electron. The *κ*_total_, *κ*_p_ and *κ*_e_ of the pure MWCNTs, PPy and MWCNTs/PPy nanocomposites were plotted as the functions of temperature and the obtained result is presented in Figure 10a–c. The values of *κ*_p_, were obtained by subtracting the values of *κ*_e_ from those of *κ*_total_.

The *κ*_total_ of the neat MWCNTs was approximately 0.16 W/mK at RT. This value increased to approximately 0.28 W/mK at 383 K (Figure 10a). These values are low compared with that reported in the literature [28]. This most probably can be attributed to the complex network, presence of defects and also to the zigzag and bamboo-like structure of the MWCNTs (Figure 1), which might have generated extra scattering sites for the phonons. The defects present in such nanotubes along with their quality were reported to be important for their thermal performance [29]. In case of the pure PPy its *κ*_total_ is found to be around 0.14 W/mK at both RT as well as at high temperature (383 K). This value is close to those reported previously [25]. The *κ*_total_ of MWCNTs seems to be temperature dependent, which is not the case for PPy. This might be due to their intrinsic as well as network structures. The MWCNTs might expand by heating and becomes closers. This close contact between the adjacent nanotubes by heating might reduce the phonons scattering sites and facilitate the phonons transport and thus increasing *κ*_total_. In case of PPy, their polymerization resulted on forming film/sheet structures; this form might not be affected by heating and could maintain the same phonons scattering sites, even if expands.

The *κ*_p_ of both MWCNTs and PPy are closer to those of *κ*_total_, and have almost the same trend with temperature, as shown in Figure 10b. This indicate that the phonons are the major heat carriers. The *κ*_e_ of both MWCNTs and PPy contributed only 3 and 4% of the *κ*_total_ of both materials, respectively. With increasing the temperature up to 383 K there is a slight increase in *κ*_e_ of both MWCNTs and PPy. This behavior with temperature is different than that of *κ*_p_ of both materials. In case of MWCNTs the *κ*_e_ is observed to be lesser temperature dependent than *κ*_p_, while in case of PPy, it is vice versa. This means PPy has electrical properties closer to perfect semiconductors than the used MWCNTs, which are impure. These MWCNTs with its considerable imperfections and low-quality graphitization exhibit poor electrical and thermal conductivities [29], but this loss in the thermal conductivity is quite useful for TE materials to have better performance.

In case of the MWCNTs/PPy nanocomposite samples (PN1–PN5), the *κ*_total_ at RT recorded higher values than those of the individuals MWCNTs and PPy (Figure 10a). The PN1 sample recorded around 0.175 W/m·K at room temperature and with increasing the MWCNTs content to 14.6 wt.% (PN5) this value increased to 0.24 W/mK. As mentioned above due to the higher amounts of catalyst particles along with complex structure of the MWCNTs, the thermal conductivity is still low in these MWCNTs/PPy nanocomposites. The included MWCNTs were used without purification and still have a considerable oxide catalyst, therefore could significantly reduce the thermal conductivity of the pure MWCNTs as well as the MWCNTs/PPy nanocomposites. The interfacing sites between the MWCNTs and PPy can also add extra scattering site for the phonons. These all tougher are responsible for this low thermal conductivity. These low thermal conductivities are useful for better figure of merit and hence TE performance. The perfect coating of the MWCNTs by PPy might facilitate the charge transport, but might also make a smooth bath for the generated phonons thus increasing the electrical conductivity. However, the MWCNTs/PPy nanocomposites still have very low thermal conductivity as compared to similar composites reported in the literature [25].

The *κ*_p_ of the MWCNTs/PPy nanocomposite samples (PN1–PN5) are closer to those of *κ*_total_, and have almost the same trend with temperature as shown in Figure 10b. This indicate that the phonons are the major heat carriers in these 1D nanocomposite samples. But, the *κ*_e_ contribution seems to be higher in these nanocomposites than in case of the individual materials. This contribution reached to around 12% of *κ*_total_. The semiconducting behavior in these 1D nanocomposite is also prominent; when the temperature increased the *κ*_e_ was observed to significantly increase (Figure 10c). With increasing the MWCNTs content in these 1D nanocomposites, the *κ*_e_ is further increased. Moreover, the rate of increases with temperature was also higher at higher MWCNTs content. This indicate that the formed 1D MWCNTs/PPy nanocomposite samples gained enhanced semiconducting properties, which might be due to the excellent 1D lamination, which promote the shipment of charges and maximize the concentration of freight easily by incorporating both MWCNTs and PPy carriers.

The perfect 1D coating of PPy on the side walls of MWCNTs significantly increased the figure of merit, *zT* of the resulting 1D core shell nanocomposites. This result is presented in in Figure 10d. The *zT* value of the neat MWCNTs was 0.7 × 10^−3^ at RT, while it slowly increased to 1.0 × 10^−3^ at 383 K. PPy showed lower *zT* values (than the pure MWCNTs), which are 0.25 × 10^−3^ at RT and 0.75 × 10^−3^ at 383 K. The *zT* values of the MWCNTs/PPy nanocomposites were significantly higher than those of both the pure MWCNTs and PPy. The highest *zT* value of 1.0 × 10^−3^ was obtained for the MWCNTs/PPy nanocomposites at all MWCNTs contents at RT. This value increased by a factor of 2.5 at 383 K for the MWCNTs/PPy nanocomposite at MWCNTs content of 4.6 wt.%.

The adopted approach in this work on perfectly coating MWCNTs with smooth layers of PPy with no PPy agglomeration and maintaining 1D core shell nanocomposites seem to be effective on improving the TE performance. The substantial increase in electrical conductivity and Seebeck coefficient resulted for this development. This improvement has been rendered rather by an important element in the wide surface area of the MWCNTs, which were used as bridges or networks linking PPy leading domains [30]. The second consideration is that MWCNTs serve as a basis for the self-assembly of PPy, which increases the electrical conductivity of the MWCNTs, in a more ordered crystalline alignment [31]. The third aspect is related to the stability and the energy filtration effect at the Seebeck coefficient values at the MWCNT/PPy interfaces. These interfaces permitted the carriers with greater energy to pass through sufficient potential limit barriers, thereby increasing the average carrier energy in the flow [25]. The low thermal conductivity of the resulting MWCNTs/PPy nanocomposites can have an important impact on dispersion of phonons due to the complex MWCNTs network and the interfaces between the MWCNT/PPy nanocomposites.

The presented results in this work demonstrated a new finding, which is the improvements in the electrical conductivity of MWCNTs by perfectly coating it with the conducting polymer, PPy to form 1D core shell nanocomposite. This finding might be a property of only conducting polymers such as PPy (using proper approach for polymerization with suitable precursors), but in case of other polymers the conductivity might decrease, as recently reported by Wang et al. [32], who mentioned that “polymer/carbon nanotube composites have lower electrical conductivity than pristine CNTs”. The latest work on coating MWCNTs with PPy has shown a strong increase in electricity conductivity [19]. The successful interaction between PPy and MWCNTs by staking the polymer chain with these MWICNTs as seen in Figure 1 could accomplish this as above stated. The connectivity between the PPy and the MWCNTs may thus be substantially strong and simple. This clear approach with the adopted simple process might be quite useful guidelines to enhance the TE performance of TE based polymer materials. It does not involve long process or complex ternary composites.

## 4. Conclusions

One dimensional core shell nanocomposite based on MWCNTs/PPy were successfully fabricated. Perfect coatings of PPy on the side walls of various concentrations of MWCNTs were performed using SDS as a surfactant. This could significantly enhance the electrical conductivity and hence the TE performance of the resulting nanocomposites. Although the used MWCNTs are impure and have considerable amounts of defects/catalysts and complex network structure, which results on a low electrical conductivity, their perfect coating with PPy significantly enhanced the electrical conductivity. The complex network structure and defects of the MWCNTs maintained the Seebeck coefficient trough energy filtering effect and also provided low thermal conductivity nanocomposites, which are essential requirements for better TE performance. This clear approach with the adopted simple process might be quite useful guidelines to enhance the TE performance of TE based polymer materials by forming perfect 1D core shell nanocomposites.

## Figures and Tables

**Table 1:** Cods and multi-walled carbon nanotubes (MWCNTs) wt.% used for different nanocomposites.

Codes	Percentage of MWCNT in the Composite (wt. %)	Composites
PN0	0	Multi-Walled Carbon Nanotubes/Polypyrrole
PN1	1.10	
PN2	2.17	
PN3	4.16	
PN4	8.00	
PN5	14.60	

## Data Availability

Not applicable.
